# Anion transport and GABA signaling

**DOI:** 10.3389/fncel.2013.00177

**Published:** 2013-10-24

**Authors:** Christian A. Hübner, Knut Holthoff

**Affiliations:** ^1^Institute of Human Genetics, Jena University Hospital, Friedrich Schiller University JenaJena, Germany; ^2^Hans Berger Department of Neurology, Jena University Hospital, Friedrich Schiller University JenaJena, Germany

**Keywords:** GABA, pH, chloride, bicarbonate, ion transporter

## Abstract

Whereas activation of GABA_A_ receptors by GABA usually results in a hyperpolarizing influx of chloride into the neuron, the reversed chloride driving force in the immature nervous system results in a depolarizing efflux of chloride. This GABAergic depolarization is deemed to be important for the maturation of the neuronal network. The concept of a developmental GABA switch has mainly been derived from *in vitro* experiments and reliable *in vivo* evidence is still missing. As GABA_A_ receptors are permeable for both chloride and bicarbonate, the net effect of GABA also critically depends on the distribution of bicarbonate. Whereas chloride can either mediate depolarizing or hyperpolarizing currents, bicarbonate invariably mediates a depolarizing current under physiological conditions. Intracellular bicarbonate is quickly replenished by cytosolic carbonic anhydrases. Intracellular bicarbonate levels also depend on different bicarbonate transporters expressed by neurons. The expression of these proteins is not only developmentally regulated but also differs between cell types and even subcellular regions. In this review we will summarize current knowledge about the role of some of these transporters for brain development and brain function.

## INTRODUCTION

GABA signaling has a wide spectrum of functions in individual neurons and neuronal networks in the brain. It is well known that in the mature brain, GABA acts as the main inhibitory transmitter due to activation of hyperpolarizing chloride currents through GABA_A_ receptors (Farrant and Kaila, 2007). In contrast, during early brain development, GABAergic transmission is assumed to provide the main excitatory drive in neuronal networks, at a time when glutamatergic synaptic contacts are less frequent than GABAergic synapses (Ben-Ari et al., 1989). Although this functional switch from excitatory to inhibitory GABA action during brain development has been observed in a wide range of preparations and different animal species, most of the experimental evidence relies on *in vitro* studies (comprehensively reviewed by Ben-Ari et al., 2007). They were performed using several electrophysiological means like intracellular recordings (Mueller et al., 1984; Luhmann and Prince, 1991) and less invasive techniques including perforated patch (Owens et al., 1996; Yamada et al., 2004) and cell-attached measurements (Wang et al., 2003; Rheims et al., 2008; Kirmse et al., 2010). Consistently, several groups reported intracellular calcium increases in immature neurons upon GABA application, most likely due to depolarization mediated activation of voltage-gated calcium channels (Yuste and Katz, 1991; Owens et al., 1996; Yamada et al., 2004; Kirmse and Kirischuk, 2006; Kirmse et al., 2010). However, *in vivo* evidence for depolarizing GABA action in immature neuronal networks is rare (Brustein et al., 2003) and often indirect (Sipila et al., 2006). Metabotropic GABA_B_-receptors are coupled to calcium or potassium channels, and cyclic AMP signaling. Although there is quite recent evidence that the non-hyperpolarizing activation of GABA_B_-receptors during development promotes neuronal migration and morphological maturation (Bony et al., 2013), this review will focus on GABA_A_-receptor signaling and how this relates to anion-transport.

The functional relevance of GABA_A_-receptor activation for activity patterns in immature neuronal networks has been investigated in different model systems *in vitro *and* in vivo*. In the immature hippocampus, it is widely accepted that GABAergic excitation drives the typical spontaneous network activity known as giant depolarization potentials or GDPs (Ben-Ari et al., 1989; Bonifazi et al., 2009). On the other hand in neocortex, glutamatergic excitation was shown to be dominating in the early generation of network activity like early network oscillations or ENOs (Garaschuk et al., 2000) and spindle-bursts (Minlebaev et al., 2007). However, more recent results suggest that GABA_A_ receptor activation also supports the generation of early neocortical network activity (Allene et al., 2008). It is worth noting that depolarizing GABA_A_-receptor activation not necessarily needs to be excitatory (Morita et al., 2005,^2006^). The GABA-induced increase in membrane conductance can also cause a so-called shunting inhibition, because according to Ohm's law, the drop in membrane resistance would decrease the voltage change caused by a certain depolarizing current (e.g., a glutamatergic synaptic input). The inhibitory effect of shunting does not depend on the polarity of a GABA-induced membrane potential change and therefore on the chloride and bicarbonate reversal potentials, but is solely due to the GABA-induced drop in input resistance. Nevertheless, the paradoxical situation could happen that a depolarizing GABA_A_-receptor activation leads to an inhibitory restriction of network activity (Minlebaev et al., 2007).

Although many initial steps in early neuronal network development are genetically determined, there is a large body of evidence, that the proper functional maturation of cortical neuronal circuits is highly activity-dependent (Katz and Shatz, 1996). However, to what extent the spontaneously occurring network activity, partly driven by GABA_A_-receptor activation, contributes to the functional maturation in the pre-sensory period of the brain is still unclear. Undoubtedly, a fine tuned balance between excitation and inhibition at any stage of development is essential for providing a proper function of neuronal networks. In this context, the potentially depolarizing mode of GABA_A_-receptor activation during early development has been considered to contribute to the higher liability to pathological events like epileptic seizures during childhood (see Kirmse et al., 2011 for review). Later in development, the increasing GABAergic inhibition governs the on- and offset of the so-called critical period in the visual system, which is crucial for the activity-dependent functional refinement of the participating cortical circuits (Hensch et al., 1998; Fagiolini and Hensch, 2000). Therefore, GABA_A_-receptor activation plays a pivotal role at various developmental stages for the maturation, refinement and proper function of neuronal networks.

The ionotropic GABA_A_ receptors are pentamers of 19 different subunits, which are grouped into eight different families according to sequence homology (α1–6, β1–3, γ1–3, δ, ε, θ, π, ρ1–3; Farrant and Kaila, 2007). Although the different receptor assemblies have different properties and different distributions, only chloride and bicarbonate are conducted under physiological conditions (Bormann et al., 1987). It is assumed that the relative bicarbonate/chloride permeability of ionotropic GABA_A_ receptors ranges between 0.18 and 0.6 (Fatima-Shad and Barry, 1993). Because of a variety of different ion transporters within the plasma membrane, which mediate acid extrusion either by extrusion of H^+^ or by accumulation of bicarbonate, the bicarbonate equilibrium potential is much more depolarized (around -10 mV) than the resting membrane potential (Roos and Boron, 1981). Hence bicarbonate can only mediate a depolarizing current under normal conditions. Quite in contrast, the equilibrium potential for chloride is close to the resting membrane potential. Hence chloride can mediate both hyperpolarizing and depolarizing currents depending on the existing gradient, which is regulated during development. However, a depolarizing action of GABA does not exclude an inhibitory action but may result in shunting inhibition as outlined above.

### ELECTROPHYSIOLOGICAL METHODS TO QUANTIFY GABA FUNCTION

Before fluorescent indicators have been available, ion-selective micro-electrodes were the gold standard for the measurement of chloride activity and pH in the intracellular compartment (Walker, 1971; Thomas, 1974; Ammann et al., 1981). Because of the invasive nature of this method – the cellular membrane needs to be impaled by a double-barreled sharp micro-electrode – it was impossible to determine the ion activity of interest quantitatively without changing it at the same time due to the measurement. Leakage currents at the site of impalement and intracellular perfusion by the solution of the reference channel are only two possible sources of measurement errors. Nevertheless, this method provided first important insights into the ionic mechanisms of pH regulation or chloride homoeostasis (Thomas, 1977; Vaughan-Jones, 1979).

In order to keep the ionic composition of the intracellular compartment unchanged, most other electrophysiological means are based on cell-attached patch clamp recordings. In the following, we describe several methods which have been developed to determine membrane potentials and membrane currents without disrupting the plasma membrane. To provide electrical access to the intracellular compartment without interference with the intracellular milieu, a technique called perforated patch clamp was developed, originally using ATP in the pipette solution as a membrane permeabilizing agent (Lindau and Fernandez, 1986). In subsequent modifications of this method, different ionophores were added to the pipette solution, which, during the experiment, incorporate into the membrane patch under the pipette tip. Ionophores are lipid-soluble molecules which form hydrophilic pores in the cell membrane and mediate electrical access to the intracellular compartment without destroying the barrier function of the membrane patch for the ion of interest. In early studies, nystatin and amphotericin B were used to achieve low resistance electrical access (Horn and Marty, 1988; Rae et al., 1991). However, as a major drawback these substances also lead to chloride redistribution. Subsequently, gramicidin D, a mixture of different antibiotics, was added because it is only permeable for monovalent cations and uncharged low molecular substances but impermeable for chloride, leaving its concentration gradient over the cell membrane intact (Ebihara et al., 1995; Kyrozis and Reichling, 1995). However, indirect changes in intracellular chloride concentration are conceivable because it is mainly regulated by cation/chloride co-transporters. Several groups have applied this method successfully to determine e.g., the chloride equilibrium potential in hippocampal cells during brain development (Mohajerani and Cherubini, 2005; Sipila et al., 2006; Tyzio et al., 2007; Pfeffer et al., 2009).

As mentioned previously, the chloride concentration gradient is not the only determinant governing GABA-induced membrane potential changes, because GABA_A_-receptor channels are also permeable for other anions like bicarbonate. In order to quantify the GABA-reversal potential directly, Tyzio et al. (2003),^2006^ developed a non-invasive method to measure the resting membrane potential and the GABA reversal potential at the same cell, using single *N*-methyl-D-aspartate and GABA channel recordings. The combination of both experimental approaches at the same cells provides the driving force for GABA-induced currents and the resting membrane potential in absolute numbers and, thereby, the GABA equilibrium potential. Although this is up to now the most reliable method to quantify these parameters, it is hardly applicable to complex preparations like *in vivo* recordings. If only the non-invasive quantification of the membrane potential change due to GABA-receptor activation is of interest, an alternative method described by Verheugen et al. (1999) can be applied (Kirmse et al., 2010). Assuming symmetrical potassium concentrations, this method uses the fact, that the reversal potential of voltage-dependent potassium currents in the cell-attached configuration represents a good estimate of the cell membrane potential (Verheugen et al., 1999). However, potential changes in intracellular potassium concentration would flaw the correct membrane potential quantification. Because a voltage-ramp protocol has to be applied for every time point of interest, the time resolution of this method is rather slow and phasic membrane potential changes are difficult to catch. In these cases, applying a current-clamp recording protocol in the cell-attached configuration might be beneficial, because it can provide a good estimate of the polarity of an induced membrane potential change at high time resolution (Perkins and Wong, 1996; Mason et al., 2005).

In summary, various non-invasive electrophysiological methods provide valuable estimates of GABA equilibrium potentials under different conditions *in vitro* and *in vivo* and enable the measurement of relative or absolute cell membrane potential changes without disturbing the intracellular milieu. In concert with complementary optical methods for quantification of intracellular chloride concentration and pH, they draw a detailed image of GABA-mediated physiological processes.

### OPTICAL METHODS TO QUANTIFY GABA FUNCTION

A big step forward was the development of fluorescent indicator dyes which enabled the optical measurement of intracellular pH and chloride concentrations (Rink et al., 1982; Illsley and Verkman, 1987). The initially used small molecular fluorescent chloride indicator dyes were quinoline derivatives which change their fluorescent intensity upon changes in chloride concentration by a mechanism called collision quenching (Chen et al., 1988). With increasing chloride concentration the probability of a collision between a chloride ion and an indicator molecule increases and therefore, its fluorescence intensity decreases by quenching. A notably feature of this mechanism is, that different from the popular calcium indicator dyes, which change their fluorescence intensity upon binding to calcium, these chloride indicator dyes do not introduce any exogenous buffer capacity to the intracellular milieu, because no binding to the ion of interest takes place. Nevertheless, their excitation spectra in the ultraviolet range give rise to strong bleaching and photodynamic damage (Inglefield and Schwartz-Bloom, 1997). However, the combination of these dyes with two-photon imaging is able to reduce both side effects significantly (Marandi et al., 2002). Several years earlier, the measurement of intracellular pH has been revolutionized by the invention of BCECF, a fluorescence indicator derived from fluorescein, by Roger Tsien and coworkers (Rink et al., 1982). The absorption spectrum of BCECF is shifted depending on changes in pH and by applying ratiometric excitation the indicator can be calibrated to absolute pH units (Graber et al., 1986; Bright et al., 1987).

The unspecific loading of the exogenously applied fluorescent indicator dyes prevents a cell-specific labeling. Therefore, much effort was invested to develop genetically determined chloride indicator dyes (see Bregestovski et al., 2009 for review). Starting point of this development was the chloride binding property of the yellow fluorescent protein (YFP) a derivative of the green fluorescent protein (GFP). Because the sensitivity of wild-type YFP to chloride is low, many random chloride binding site mutations of YFP were tested and analyzed for improved sensitivity (Galietta et al., 2001). Besides the possibility of cell-specific expression of the chloride indicator, YFP-based indicator dyes have additional advantages. Different from quinolone-derived dyes, the optimal excitation wavelength is located in the visible range, providing less bleaching and photodynamic damage during the experiments. In addition, leakage during the measurements is less pronounced due to their relative large molecular weight of about 27 kDa (Bregestovski et al., 2009). Finally and different from calcium measurements, intracellular indicator concentrations are orders of magnitude smaller than that of the ion of interest, therefore exogenous buffering of chloride is negligible. On the other hand, there are also some disadvantages of YFP-based indicator dyes. Keeping in mind that changes in intracellular chloride concentration are often accompanied by changes in pH, the significant pH-sensitivity of many YFP derivatives is the most serious one. The only way to circumvent this restriction is the independent monitoring of pH changes and subsequent data correction. Another problem of YFP-based chloride indicator dyes are their rather slow kinetics or poor sensitivity, which either limits the detection of fast chloride concentration changes or leads to poor resolution at physiological chloride levels (Galietta et al., 2001). Originally, YFP-based indicator dyes were not able to report absolute levels of chloride concentration, because they lack an isosbestic point, at which they are insensitive to chloride concentration changes. Because the absolute measurement of intracellular chloride concentrations is imperative to determine the chloride equilibrium potential or the driving force for chloride, Kuner and Augustine (2000) developed a ratiometric chloride indicator named Clomeleon. Clomeleon uses the chloride-dependent interaction of two fluorophores (cyan fluorescent protein (CFP) as donor and a variant of YFP called topas fluorescent protein (TFP) as acceptor) by Förster energy transfer (FRET). Upon chloride binding to TFP, the efficiency of FRET between CFP and TFP declines. As a consequence, the ratio of TFP and CFP fluorescence emission drops with increasing chloride concentrations. Because the emission spectrum comprises an isosbestic point, calibration to absolute chloride levels is possible (Kuner and Augustine, 2000). Unfortunately, the sensitivity of Clomeleon with an IC_50_ of more than 160 mM is rather low and, at physiological levels, makes reliable measurements of absolute intracellular chloride concentration very difficult (Kuner and Augustine, 2000). Following genetic engineering of the YFP chloride binding site yielded a higher sensitivity of the resulting indicator called Cl-sensor with an IC_50_ around 30 mM, much closer to physiological intracellular chloride concentrations (Markova et al., 2008). However, Clomeleon and Cl-sensor have slow response kinetics and share the pH sensitivity of all YFP-based chloride indicators (Bregestovski et al., 2009).

To overcome the main drawbacks of Clomeleon and Cl-sensor, a new ratiometric but non-FRET-based sensor was developed (Arosio et al., 2010). This new indicator, called ClopHensor, is suitable for the simultaneous quantification of intracellular pH and chloride concentration. Therefore, a variant of enhanced GFP (E^2^GFP) with pH sensitivity and sensitivity to chloride comparable to Cl-sensor was fused with a pH- and chloride-insensitive monomeric DsRed. The E^2^GFP part of the fused protein allows chloride-independent ratiometric quantification of pH by exciting it subsequently at 458 and 488 nm. The ratiometric measurement of chloride concentration by alternative exciting E^2^GFP at 458 nm and the chloride-insensitive DsRed at 543 nm requires in addition the calibration at different pH values. However a simultaneous quantification of intracellular pH is now possible, ClopHensor still suffers from rather low sensitivity to chloride with an IC_50_ around 40 mM (Arosio et al., 2010; Mukhtarov et al., 2013). A more recent variant of ClopHensor exhibits a higher sensitivity with an IC_50_ of 20 mM, but at the expense of a significant lower dynamic range (Mukhtarov et al., 2013). In summary, the development of the ratiometric indicator ClopHensor provides a most valuable means for the simultaneous quantification of pH and chloride concentration, but variants with higher sensitivity to chloride are desired to increase the quantification accuracy at physiological concentration levels.

## ION TRANSPORTERS INVOLVED IN THE REGULATION OF NEURONAL CHLORIDE AND BICARBONATE LEVELS

**FIGURE 1:**
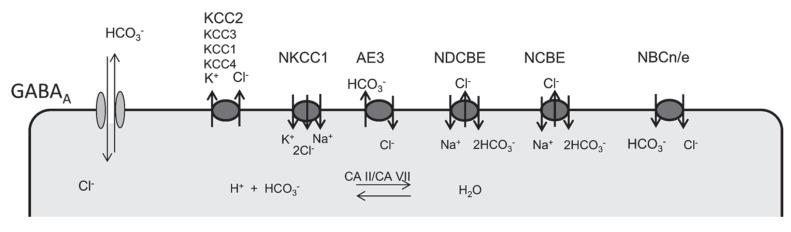
**Ion transporters involved in the regulation of neuronal chloride and bicarbonate levels.** GABA receptors are permeable for both chloride and bicarbonate. Several anion transporters are expressed in neurons and may thus also affect GABA signaling. Whereas NKCC1 is the main chloride accumulating transporter in most neurons, KCC2 is the most important chloride extruder. Anion-transporters of the SLC4A family of bicarbonate transporters can be sub-classified in Na^+^ dependent (NDCBE, NCBE) and Na^+^ independent anion exchangers (AE1,2,3). According to the stoichiometry Na^+^-coupled bicarbonate co-transports can be either electroneutral (NBCn) or electrogenic (NBCe). Although carbonic anhydrases cannot change the existing bicarbonate gradients, they promote anion-transport by members of the SlC4A family and replenish bicarbonate levels.

The role of cation-chloride co-transporters (**Figure 1**) in the regulation of the intraneuronal chloride concentration has been extensively studied and follows a well-defined developmental sequence with a high chloride concentration in immature neurons due to neuronal chloride accumulation. Chloride accumulation largely depends on the action of the Na^+^/K^+^/2Cl^-^ co-transporter NKCC1 (Yamada et al., 2004; Sipila et al., 2006; Achilles et al., 2007; Blaesse et al., 2009; Pfeffer et al., 2009). But other mechanisms to accumulate chloride exist and maintain GABA depolarizing even in the absence of NKCC1 (Pfeffer et al., 2009). One candidate is the anion-exchanger AE3, which normally accumulates chloride in exchange for intracellular bicarbonate and thereby raises intracellular chloride levels. The so-called GABA switch from excitatory to inhibitory is brought about by the incipient expression of the cation-chloride co-transporter KCC2 (Rivera et al., 1999; Hübner et al., 2001; Stein et al., 2004), which extrudes chloride out of the cell. From our knockout studies other KCl co-transporters like KCC1 (Rust et al., 2007), KCC3 (Boettger et al., 2003; Seja et al., 2012), or KCC4 (Boettger et al., 2002) appear to be less important in the control of neuronal chloride levels.

Several transporters either exchange bicarbonate and chloride or couple the transport of bicarbonate to sodium (**Figure 1**). For bicarbonate transporters that also transport chloride, the net effect for GABA_A_ receptor signaling is difficult to predict. It is evident that changes of bicarbonate levels do not only directly affect the currents mediated by GABA_A_ receptors, but are also tightly linked to alterations of the pH both within the cells and in the extracellular space, which can have a multitude of different effects. In neurons with a high intracellular chloride concentration as in the immature nervous system, however, the effect of bicarbonate on the GABA_A_ reversal potential is quite low according to the Goldman equation (Farrant and Kaila, 2007).

### ANION EXCHANGERS

**FIGURE 2:**
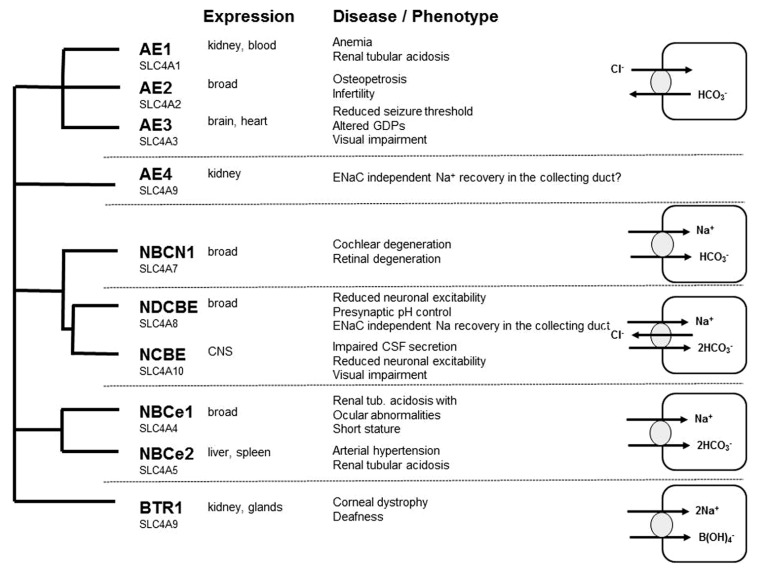
**Overview of the SLC4A family of bicarbonate transporter: expression, loss of function phenotypes, and transport characteristics**.

Whereas there are numerous reviews addressing the role of cation-chloride co-transporters for GABA transmission (Blaesse et al., 2009), the role of bicarbonate and hence the role of neuronal mechanisms to control intracellular bicarbonate levels are less acknowledged. Bicarbonate transport is mediated by members of the SLC4A or the SLC26A family of proteins. As members of the SLC26A family appear to play a minor role for neurons (Dorwart et al., 2008; Majumdar and Bevensee, 2010), we restrict our review to some selected members of the SLC4A family with known relevance for neuronal function and refer to some other more complete reviews (Romero et al., 2004; Dorwart et al., 2008; Majumdar and Bevensee, 2010). The SLC4 family can be subdivided into four main branches: the sodium independent anion-exchangers (AE1, AE2, and AE3) recently reviewed in Alper (2009), and sodium-coupled bicarbonate transporters recently reviewed in Majumdar and Bevensee (2010; **Figure 2**). The role of AE4 is still unclear: although originally cloned as a sodium-independent anion-exchanger, there is evidence that it rather serves as a Na^+^/HCO_3_^-^ co-transporter (Parker et al., 2002). It localizes highly specific to the basolateral membrane of mouse type B intercalated cells and is involved in chloride recovery by these cells (Chambrey et al., 2013). Na^+^/HCO_3_^-^ co-transporters can be either electroneutral (NBCn1 and NBCn2) or electrogenic (NBCe1 and NBCe2), whereas the sodium-dependent anion-exchangers (NDAE or NDCBE and NCBE) are electroneutral. SLC4A11 differs from the other family members because it rather mediates borate transport and is hence termed as BTR1 (Park et al., 2004). In the following we will focus on AE3, NCBE, and NDCBE which are strongly expressed in the brain.

#### AE3

In nervous tissue, the AE3 transporter has been localized to neurons (Kopito et al., 1989; Raley-Susman et al., 1993), and to Muller cells and horizontal neurons within the retina (Kobayashi et al., 1994). In many neurons, anion-exchange is thought to be mainly mediated by AE3 (Kopito et al., 1989; Hentschke et al., 2006; Romero et al., 2013). In contrast, AE1 plays an important role for bicarbonate recovery of intercalated cells in the kidney and for red blood cells, where it is one of the most abundant proteins of the plasma membrane (band3). Accordingly, mutations in AE1 can cause renal tubular acidosis and/or hemolytic anemia (Alper, 2009). AE2 is the most widely expressed anion-exchanger, which localizes to the basolateral side in most epithelial cells (Romero et al., 2013). Like AE1 it appears to play a minor role for the control of the neuronal pH. The *SLC4A3* gene encoding AE3 employs two different promoters to generate the brain and the cardiac variant, the latter having a shorter amino-terminal amino acid sequence. Because of its broad neuronal expression, the brain variant of AE3 is also often referred to as the neuronal anion-exchanger. Transcripts were already detected at early developmental stages of murine brain development starting around E11 (Hentschke et al., 2006). Because of its early expression and its predicted role to raise the intracellular chloride concentration above the electrochemical equilibrium, AE3 may also contribute to early GABAergic excitation. In particular, it has been hypothesized that AE3 is responsible for chloride accumulation in lateral superior olivary neurons at P0–P3 (Becker et al., 2003). During this time window, these neurons express AE3 but not NKCC1 and depolarize in response to glycine (Balakrishnan et al., 2003). Surprisingly, however, anion-exchange was nearly absent from cultured fetal neurons, although AE3 mRNA was found in both fetal and adult hippocampal neurons (Raley-Susman et al., 1993).

**FIGURE 3:**
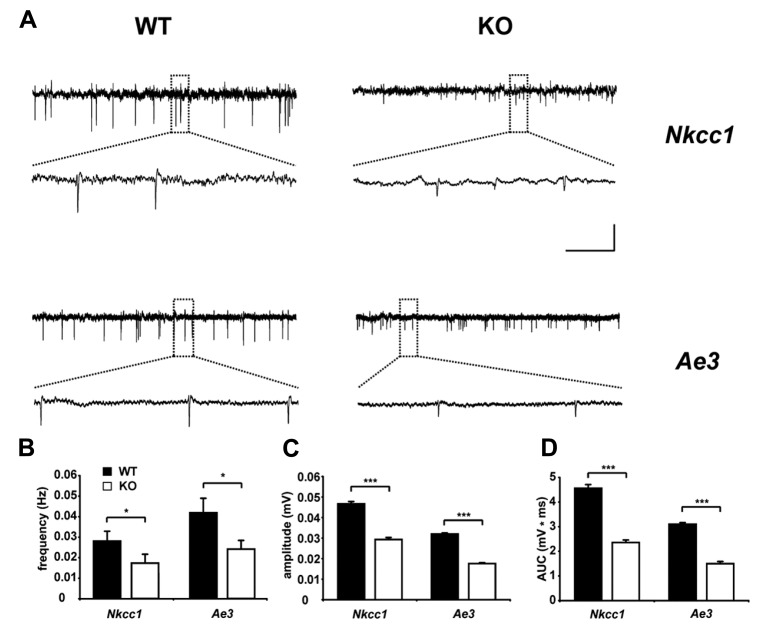
**Reduced spontaneous electrical activity in hippocampal slices of NKCC1 and AE3 knockout mice.** **(A)** Representative extracellular recordings from the stratum pyramidale (CA3) of postnatal day 5 NKCC1 and AE3 WT and KO slices. The framed parts are shown as enlargements below the original trace. Calibration: horizontal, 2 min (original trace), 7.5 s (enlargement); vertical, 0.04 mV. Quantification of frequency **(B)**, amplitude **(C)**, area under curve (AUC), and **(D)** of single spontaneous electrical events. The asterisks indicate significant difference (*p < 0.05, ***p < 0.001, *t*-test). Error bars indicate SEM. Reprinted from Pfeffer et al. (2009).

Interestingly, AE3 expression levels in cultured hippocampal neurons from rat increased during long-term exposure to ammonia and caused an ammonia induced increase of the intracellular chloride concentration (Irie et al., 1998), thus supporting a role of AE3 for the regulation of the intraneuronal chloride concentration. At the protein level, a clear band corresponding to AE3, which was absent from knockout tissues, was detected in murine P1 brain lysates with increasing signal intensities at P5 and P15 (Pfeffer et al., 2009). Unfortunately, the subcellular localization of AE3 in the brain is still unclear, because no antibody has been reported that reliably detects endogenous AE3 in brain sections. The GABA reversal potential and GABA-evoked Ca^2^^+^ responses of CA1 neurons of AE3 knockout mice did not differ between AE3 knockout and WT mice at P1 (Pfeffer et al., 2009), suggesting that in this type of neuron at this time point chloride accumulation by AE3 may be marginal compared to NKCC1. Nevertheless, this may change with increasing expression levels of AE3 during brain maturation. Supporting that AE3 modulates GABAergic transmission, similar to NKCC1 knockout mice GDPs, which largely depend on a depolarizing action of GABA (Leinekugel et al., 1997; Ben-Ari et al., 2007), were reduced in terms of frequency and amplitudes at postnatal day 5 in AE3 knockout mice (**Figure 3**; Pfeffer et al., 2009), but these changes may also be related to changes in neuronal pH homeostasis. Although the intraneuronal pH at steady-state conditions in principal neurons of the adult mouse hippocampus did not differ between genotypes, the recovery from an alkaline load was drastically reduced in neurons devoid of AE3 (Hentschke et al., 2006). Hence, the role of AE3 for chloride accumulation in hippocampal neurons should be re-addressed at later developmental stages and in different types of neurons. Indeed, in spinal cord motoneurons chloride accumulation was in part bicarbonate-dependent and sensitive to anion-exchange blockers (Gonzalez-Islas et al., 2009). These findings are in accordance with a previous report on GABA currents in embryonic motoneurons, which were dampened by bumetanide and removal of extracellular bicarbonate (Kulik et al., 2000). It has been estimated that NKCC1 is responsible for approximately two-thirds of the steady-state chloride accumulation, whereas AE3 for the remaining third (Gonzalez-Islas et al., 2009). NKCC1 and AE3 may thus have distinct functions in the recovery of chloride levels following chloride depletion in embryonic motoneurons.

Overall, no obvious behavioral or morphological alterations of the brain of AE3 knockout mice have been reported (Hentschke et al., 2006; Alvarez et al., 2007). Notably, the seizure threshold in response to various proconvulsive agents was significantly reduced upon disruption of AE3 (Hentschke et al., 2006). This observation supports a previous report that a susceptibility locus for common idiopathic generalized epilepsy maps to chromosomal region 2q36 (Sander et al., 2002), which also includes *SLC4A3*. Indeed, in a subsequent study a common polymorphism within the coding sequence of *SLC4A3*, which entails the amino acid exchange Ala867Asp, was associated with an increased risk to develop idiopathic generalized epilepsy (Sander et al., 2002). Moreover, the Ala867Asp variant had a significantly reduced anion-exchange activity compared to wild-type in a heterologous expression system, whereas differences in expression levels or protein trafficking to the plasma membrane were excluded (Vilas et al., 2009). Nevertheless, it is still unclear whether the above-mentioned polymorphism 867Asp itself confers the increased risk for epileptic seizures or another gene in close proximity of *SLC4A3* is involved.

Inner retina defects with late onset photoreceptor degeneration with optic nerve and retinal vessel anomalies, which resulted in reduction of the b-wave in electroretinograms, were noted in an independent AE3 knockout mouse (Alvarez et al., 2007). In the retina, the brain variant of AE3 localized to Müller cells, whereas the cardiac variant was detected in horizontal cells. Immuno-labeling of astrocytes showed that inner retina vessels were wrapped by dense astrocytic processes at 8 months of age in AE3 knockout mice. Moreover, inner retina blood vessels formed sporadic loops in the knockout, a finding which was not observed in wild-type mice. Immunoblotting analysis revealed that the Na^+^/HCO_3_^-^ co-transporter (NBC1), and carbonic anhydrases (CAs) II and CA XIV protein expression were elevated in AE3 knockout mice mouse retinas, suggesting a partial compensation for loss of AE3. Anion-exchange activity mediated by AE3 is promoted by the action of extracellular CAs (Svichar et al., 2009). AE3 associates with the CAs and forms a bicarbonate transport metabolon to maximize bicarbonate fluxes across the plasma membrane (Casey et al., 2009).

#### Sodium-coupled anion exchangers

Historically, sodium-dependent anion-exchange (NDAE) which extrudes chloride from cells was the first acid–base transport mechanism described to play a role in the control of intracellular pH (Boron and De Weer, 1976). A cDNA encoding a protein that mediates NDAE (also referred to as NDCBE) was initially cloned from *Drosophila* (Romero et al., 2000). The mRNA was expressed throughout *Drosophila* development with a prominent signal in the central nervous system and its disruption resulted in a lethal phenotype in *Drosophila*. A related cDNA coding for another protein mediating NCBE was cloned from a mouse insulinoma cell line (Wang et al., 2000). This initial transport characterization was subsequently confirmed for rat (Giffard et al., 2003; Damkier et al., 2010), whereas the human cDNA was rather characterized as an electroneutral Na^+^/HCO_3_^-^ cotransporter (NBCn2) with chloride self-exchange activity (Parker et al., 2008b). Some of the controversy may be explained by the different expression systems used in the different studies like mammalian cells and *Xenopus* oocytes, temperature, and composition of solutions, the transfection/injection efficiency or molecular tagging of the transport proteins.

Sodium-coupled anion exchange is activated by intracellular acidification (Schwiening and Boron, 1994), suggesting that regulation of the chloride gradient by NDAEs may be closely linked to the regulation of cellular pH. As prolonged neuronal activity can cause neuronal acidification by efflux of bicarbonate through GABA_A_ receptors (Kaila and Voipio, 1987), sodium-coupled anion exchange may help to maintain a hyperpolarizing chloride reversal potential and thus promote the inhibitory action of GABA. Thus activation of sodium-coupled anion exchange by acidosis may also contribute to seizure termination by promoting a more negative chloride reversal potential and thus promoting the inhibitory effects of GABA.

***NDCBE***. Several transcript variants have been reported for human and murine NDCBE. The functional comparison of the NDCBE variants expressed in *Xenopus* oocytes demonstrated that the variants with a shorter C-terminus had a reduced functional expression, whereas the different amino termini did not affect the basal functional expression of NDCBE (Parker et al., 2008a). NDCBE is encoded by *SLC4A8* and is broadly expressed in different tissues including brain (Romero et al., 2004). A down-regulation of NDCBE protein expression was shown in different brain regions after chronic hypoxia with a different profile in neonates and adult mice (Chen et al., 2008a). Immunoreactivity for NDCBE was detected in different brain regions with no overlap to astrocyte markers (Chen et al., 2008b). This was also confirmed in a knockout controlled study with an independent polyclconal antibody against NDCBE. Moreover, this study reported that NDCBE localization overlapped with markers of presynaptic glutamatergic but not GABAergic nerve terminals (Sinning et al., 2011). From Western analysis of different brain lysate subfractions and immunogold electron microscopy studies on isolated synaptosomes, it was further concluded that NDCBE is enriched in presynaptic nerve endings of excitatory neurons. The localization in presynaptic glutamatergic terminals was also shown in an independent study, but in contrast to the previous report the latter study also detected NDCBE in terminals of parvalbumin-positive GABAergic cells (Burette et al., 2012). Hence, the authors speculated that NDCBE may play a role as a regulator of GABAergic neurotransmission.

Confirming the important role of NDCBE for pH regulation in neurons, its disruption caused a sustained decrease of the steady-state pH of cultured hippocampal neurons (Sinning et al., 2011). In accordance with the observation that NDCBE co-localizes with presynaptic glutamatergic nerve terminals, the frequency of miniature excitatory postsynaptic currents (mEPSCs) was drastically reduced in a pH-dependent manner in hippocampal neurons of mice devoid of NDCBE, whereas miniature inhibitory postsynaptic currents (mIPSCs) were unchanged. Importantly, the effect on mEPSCs could be at least in part restored by shifting the pH, strongly arguing against a structural defect (Sinning et al., 2011). Its role during early brain maturation, however, has not been studied.

Whether NDCBE also significantly contributes to the control of the intraneuronal chloride concentration in some neurons is still unclear. It was observed that dopaminergic neurons in the rat substantia nigra do not express KCC2, but still exhibit inhibitory responses to GABA that are dependent upon the presence of extracellular bicarbonate (Gulacsi et al., 2003). As the GABA reversal potential was significantly less negative in bicarbonate-free buffer in dopaminergic neurons, a sodium-dependent anion might substitute KCC2 in this type of neuron. This assumption was also supported by the demonstration that complex-spike activity in some auditory interneurons results in a pH-dependent negative shift of the glycine reversal potential, and it was suggested that sodium-coupled anion exchange via Slc4a8 may account for the reduction of intracellular chloride (Kim and Trussell, 2009).

In *Caenorhabditis elegans* sodium-coupled anion exchange is mediated by ABTS-1. While animals lacking ABTS-1 or KCC2 displayed only mild behavioral defects, disruption of both chloride extruders resulted in a paralytic phenotype (Bellemer et al., 2011). Although direct electrophysiological data were not provided, the authors speculated that the disruption of both transporters results in a reversal of chloride fluxes through GABA_A_ receptors thus rather exciting than inhibiting cells. Moreover, neuronal expression of both transporters was up-regulated during neuronal differentiation and ABTS-1 expression was increased in mutants devoid of KCC2, suggesting that both transporters are important to control the cellular chloride gradient.

***NCBE***. For NCBE two different splice variants have been identified with different expression profiles (Giffard et al., 2003). The variant missing a 39-bp insert at the 3^′^ end is predicted to result in a protein with a C-terminal PDZ motif (Giffard et al., 2003). How this might relate to function has not been studied. Our expression analysis in the developing mouse brain with a probe detecting both transcript variants revealed a broad neuronal expression pattern and a particularly strong labeling of the choroid plexus (Hübner et al., 2004). At the protein level, NCBE localized to the basolateral membrane of choroid plexus epithelial cells (Jacobs et al., 2008). There, NCBE serves as a basolateral sodium entry pathway. According to this model, its disruption is predicted to impair cerebrospinal fluid secretion, which is supported by the finding that mice with a targeted disruption of NCBE display a collapse of their brain ventricles (Jacobs et al., 2008). Immunohistological studies revealed that the NCBE protein mainly localized to dendrites and somata of principal neurons, but not to axons or astrocytes (Chen et al., 2008b; Jacobs et al., 2008). There was also a considerable overlap between GABAergic interneurons as identified by the co-localization of GAD and NCBE (Jacobs et al., 2008). However, to which extent NCBE can be detected in different interneuron subtypes, still remains to be addressed. Notably, the ultrastructural analysis also localized NCBE preferentially to dendrites and spines both in the hippocampus as well as in the cerebellum (Jacobs et al., 2008).

Although there was no difference in the steady-state pH of principal neurons of the CA1 hippocampal region of NCBE knockout mice (Jacobs et al., 2008), the recovery to an acid load was delayed. How this affects network excitability was studied in the 4-aminopyridine model of interictal discharges in acute brain slices. The frequency of the interictal-like events at baseline levels did not differ between genotypes, however, the decreased frequency upon a propionate pulse was prolonged in the knockout. In accordance, knockout mice had an increased seizure threshold in response to different seizure inducing agents including pentylenetetrazole or pilocarpine. Quite in contrast to the mouse findings, in some patients with epilepsy larger heterozygous genomic deletions involving *SLC4A10* were described (McMilin et al., 1998), however, the genetic evidence that the epilepsy phenotype is directly linked to the heterozygous loss of NCBE or rather to some other genes within this chromosomal region is obscure. Because of the different sites of NCBE expression both in excitatory and inhibitory neurons as well as in the choroid plexus different effects may add up in the total knockout. Thus, the exact role of NCBE for network excitability and synaptic transmission still needs to be addressed in more specific mouse models.

NCBE is also strongly expressed within the retina, where it localizes to ON and OFF bipolar cell axon terminals and to dendrites of OFF bipolar cells, where it co-localized with the main neuronal chloride extruder KCC2 (Hilgen et al., 2012). NCBE was also expressed in starburst amacrine cells, but was absent from neurons known to depolarize in response to GABA, like horizontal cells. These data suggest that NCBE may indeed contribute to the regulation of intracellular chloride and bicarbonate concentration in retinal neurons. Supporting this assumption, knockout mice displayed a decreased visual acuity and contrast sensitivity in behavioral experiments and smaller b-wave amplitudes and longer latencies in electroretinograms (Hilgen et al., 2012).

## CARBONIC ANHYDRASES

In the mature rat hippocampus, intense GABA_A_ receptor activation causes neuronal excitation which is strictly dependent on the presence of bicarbonate and suppressed by membrane-permeant inhibitors of CA activity (Staley et al., 1995; Kaila et al., 1997; Fujiwara-Tsukamoto et al., 2007). Fifteen members of the CA family have been identified which differ in tissue distribution and subcellular localization. At least 13 family members catalyze the reversible hydration of CO_2_ to form bicarbonate and H^+^, accelerating this spontaneous reaction several thousand-fold. Thereby CAs influence the kinetics and amplitudes of pH transients in distinct intra- and extracellular compartments (Chesler, 2003; Casey et al., 2009) and can affect proton-sensitive membrane proteins involved in neuronal signaling such as GABA_A_ receptors, NMDA receptors, and many more. CA also associate with anion exchangers to form bicarbonate transport metabolons, which enhance bicarbonate fluxes across the plasma membrane (McMurtrie et al., 2004). By forming isoform-specific metabolons with distinct acid–base transporters intracellular neuronal CAs may contribute to developmentally and spatially distinct pH*_i_* microdomains. In the brain, extracellular space CA activity is due mainly to isoforms CA IV and CA XIV, which both play important roles in the regulation of intracellular pH in hippocampal neurons by facilitating AE3-mediated Cl^-^/HCO_3_^-^ exchange (Casey et al., 2009; Svichar et al., 2009).

CA II and CA VII are the only cytosolic isoforms present in both somata and dendrites of mature hippocampal CA1 pyramidal neurons. The functional expression of CA VII in mouse brain starts around postnatal day 10 (P10) and that of CA II around P20 and coincides with the appearance of bicarbonate-dependent high frequency stimulation (HFS)-induced tonic GABAergic excitation (Ruusuvuori et al., 2004,^2013^). Synchronous neuronal activity in the form of GDPs, however, starts much earlier with an onset at approximately P0 (Ben-Ari et al., 1989) and is largely independent from the presence of bicarbonate (Ruusuvuori et al., 2004). These GDPs disappear with the on-going expression of the chloride extruding K^+^–Cl^-^ cotransporter KCC2 which is up-regulated from P0 to P12 thus rendering GABA_A_ responses hyperpolarizing (Rivera et al., 1999; Hübner et al., 2001; Stein et al., 2004). To study the role for cytoplasmic neuronal CAs for bicarbonate-dependent GABAergic depolarization, we recently established a CA VII knockout mouse model. Remarkably, CA VII knockout mice have a normal life span and show no gross behavioral abnormalities. At P13–14, when CA II is not yet expressed, CA VII KO mice show a complete absence of electrographic seizures (Ruusuvuori et al., 2013). These results point to a crucial role for the developmental expression of intrapyramidal CAs in shaping integrative functions, long-term plasticity and susceptibility to epileptogenesis and put intraneuronal CA in a key position in GABAergic excitation (Kaila et al., 1997; Ruusuvuori et al., 2004). Moreover, these observations give important insights into the antiepileptic actions of CA inhibitors.

## CONCLUSION

There is ample evidence that brain development and brain function critically depends on anion gradients. Whereas chloride has been in the focus of the neuroscientific community, much less is known about bicarbonate. With the development of several mouse models with targeted disruption of selected players of intraneuronal bicarbonate levels, some described in this review, first clues how bicarbonate contributes to proper brain function like the production of the cerebrospinal fluid, neuronal excitability, and synaptic transmission evolved. The role of these processes for brain development is mostly unknown but it emerges that bicarbonate transporters modulate GABAergic transmission already in the developing brain. It will be essential to assess whether this reflects secondary effects in response to changes in pH or whether these effects rather reflect alterations of the existing anion gradients. Bicarbonate definitely plays an essential role for the GABAergic excitation observed upon massive GABAergic stimulation. This process is massively enhanced by CAs, which quickly replenish intraneuronal bicarbonate from P18 onwards. To understand the complex interplay of the different proteins in time and space is an emerging challenge for the future.

## Conflict of Interest Statement

The authors declare that the research was conducted in the absence of any commercial or financial relationships that could be construed as a potential conflict of interest.

## References

[B1] Achilles K. Okabe A. Ikeda M. Shimizu-Okabe C. Yamada J. Fukuda A. (2007). Kinetic properties of Cl uptake mediated by Na^+^-dependent K^+^–2Cl cotransport in immature rat neocortical neurons. *J. Neurosci.* 27 8616–8627 10.1523/JNEUROSCI.5041-06.2007 17687039 PMC6672936

[B2] Allene C. Cattani A. Ackman J. B. Bonifazi P. Aniksztejn L. Ben-Ari Y. (2008). Sequential generation of two distinct synapse-driven network patterns in developing neocortex. *J. Neurosci.* 28 12851–12863 10.1523/JNEUROSCI.3733-08.2008 19036979 PMC6671804

[B3] Alper S. L. (2009). Molecular physiology and genetics of Na^+^-independent SLC4 anion exchangers. *J. Exp. Biol.* 212 1672–1683 10.1242/jeb.029454 19448077 PMC2683012

[B4] Alvarez B. V. Gilmour G. S. Mema S. C. Martin B. T. Shull G. E. Casey J. R. (2007). Blindness caused by deficiency in AE3 chloride/bicarbonate exchanger. *PLoS ONE *2:e839. 10.1371/journal.pone.0000839 PMC1950688 17786210

[B5] Ammann D. Lanter F. Steiner R. A. Schulthess P. Shijo Y. Simon W. (1981). Neutral carrier based hydrogen ion selective microelectrode for extra- and intracellular studies. *Anal. Chem.* 53 2267–2269 10.1021/ac00237a031 7316213

[B6] Arosio D. Ricci F. Marchetti L. Gualdani R. Albertazzi L. Beltram F. (2010). Simultaneous intracellular chloride and pH measurements using a GFP-based sensor. *Nat. Methods* 7 516–518 10.1038/nmeth.1471 20581829

[B7] Balakrishnan V. Becker M. Lohrke S. Nothwang H. G. Guresir E. Friauf E. (2003). Expression and function of chloride transporters during development of inhibitory neurotransmission in the auditory brainstem. *J. Neurosci.* 23 4134–4145 12764101 10.1523/JNEUROSCI.23-10-04134.2003 PMC6741087

[B8] Becker M. Nothwang H. G. Friauf E. (2003). Differential expression pattern of chloride transporters NCC, NKCC2, KCC1, KCC3, KCC4, and AE3 in the developing rat auditory brainstem. *Cell Tissue Res.* 312 155–165 12712325 10.1007/s00441-003-0713-5

[B9] Bellemer A. Hirata T. Romero M. F. Koelle M. R. (2011). Two types of chloride transporters are required for GABA(A) receptor-mediated inhibition in *C. elegans.* *EMBO J.* 30 1852–1863 10.1038/emboj.2011.83 21427702 PMC3101993

[B10] Ben-Ari Y. Cherubini E. Corradetti R. Gaiarsa J. L. (1989). Giant synaptic potentials in immature rat CA3 hippocampal neurones. *J. Physiol.* 416 303–325 2575165 10.1113/jphysiol.1989.sp017762 PMC1189216

[B11] Ben-Ari Y. Gaiarsa J. L. Tyzio R. Khazipov R. (2007). GABA: a pioneer transmitter that excites immature neurons and generates primitive oscillations. *Physiol. Rev.* 87 1215–1284 10.1152/physrev.00017.2006 17928584

[B12] Blaesse P. Airaksinen M. S. Rivera C. Kaila K. (2009). Cation-chloride cotransporters and neuronal function. *Neuron* 61 820–838 10.1016/j.neuron.2009.03.003 19323993

[B13] Boettger T. Hübner C. A. Maier H. Rust M. B. Beck F. X. Jentsch T. J. (2002). Deafness and renal tubular acidosis in mice lacking the K-Cl co-transporter Kcc4. *Nature* 416 874–878 10.1038/416874a 11976689

[B14] Boettger T. Rust M. B. Maier H. Seidenbecher T. Schweizer M. Keating D. J. (2003). Loss of K–Cl co-transporter KCC3 causes deafness, neurodegeneration and reduced seizure threshold. *EMBO J.* 22 5422–5434 10.1093/emboj/cdg519 14532115 PMC213773

[B15] Bonifazi P. Goldin M. Picardo M. A. Jorquera I. Cattani A. Bianconi G. (2009). GABAergic hub neurons orchestrate synchrony in developing hippocampal networks. *Science* 326 1419–1424 10.1126/science.1175509 19965761

[B16] Bony G. Szczurkowska J. Tamagno I. Shelly M. Contestabile A. Cancedda L. (2013). Non-hyperpolarizing GABAB receptor activation regulates neuronal migration and neurite growth and specification by cAMP/LKB1. *Nat. Commun.* 4 1800 10.1038/ncomms2820 23653212

[B17] Bormann J. Hamill O. P. Sakmann B. (1987). Mechanism of anion permeation through channels gated by glycine and gamma-aminobutyric acid in mouse cultured spinal neurones. *J. Physiol.* 385 243–286 2443667 10.1113/jphysiol.1987.sp016493 PMC1192346

[B18] Boron W. F De Weer P. (1976). Intracellular pH transients in squid giant axons caused by CO_2_, NH_3_, and metabolic inhibitors. *J. Gen. Physiol.* 67 91–112 10.1085/jgp.67.1.91 1460 PMC2214912

[B19] Bregestovski P. Waseem T. Mukhtarov M. (2009). Genetically encoded optical sensors for monitoring of intracellular chloride and chloride-selective channel activity. *Front. Mol. Neurosci. *2:15. 10.3389/neuro.02.015.2009 PMC2802328 20057911

[B20] Bright G. R. Fisher G. W. Rogowska J. Taylor D. L. (1987). Fluorescence ratio imaging microscopy: temporal and spatial measurements of cytoplasmic pH. *J. Cell Biol.* 104 1019–1033 10.1083/jcb.104.4.1019 3558476 PMC2114443

[B21] Brustein E. Marandi N. Kovalchuk Y. Drapeau P. Konnerth A. (2003). “*In vivo*” monitoring of neuronal network activity in zebrafish by two-photon Ca^2^^+^ imaging. *Pflugers Arch.* 446 766–773 10.1007/s00424-003-1138-4 12883893

[B22] Burette A. C. Weinberg R. J. Sassani P. Abuladze N. Kao L. Kurtz I. (2012). The sodium-driven chloride/bicarbonate exchanger in presynaptic terminals. *J. Comp. Neurol.* 520 1481–1492 10.1002/cne.22806 22102085 PMC3856893

[B23] Casey J. R. Sly W. S. Shah G. N. Alvarez B. V. (2009). Bicarbonate homeostasis in excitable tissues: role of AE3 Cl^-^/HCO_3_^-^ exchanger and carbonic anhydrase XIV interaction. *Am. J. Physiol. Cell Physiol.* 297 C1091–C1102 10.1152/ajpcell.00177.2009 19692653 PMC2777400

[B24] Chambrey R. Kurth I. Peti-Peterdi J. Houillier P. Purkerson J. M. Leviel F. (2013). Renal intercalated cells are rather energized by a proton than a sodium pump. *Proc. Natl. Acad. Sci. U.S.A.* 110 7928–7933 10.1073/pnas.1221496110 23610411 PMC3651478

[B25] Chen L. M. Haddad G. G. Boron W. F. (2008a). Effects of chronic continuous hypoxia on the expression of SLC4A8 (NDCBE) in neonatal versus adult mouse brain. *Brain Res.* 1238 85–92 10.1016/j.brainres.2008.08.033 18775686 PMC3222586

[B26] Chen L. M. Kelly M. L. Parker M. D. Bouyer P. Gill H. S. Felie J. M. (2008b). Expression and localization of Na-driven Cl^-^/HCO_3_^-^ exchanger (SLC4A8) in rodent CNS. *Neuroscience* 153 162–174 10.1016/j.neuroscience.2008.02.018 18359573 PMC2905791

[B27] Chen P. Y. Illsley N. P. Verkman A. S. (1988). Renal brush-border chloride transport mechanisms characterized using a fluorescent indicator. *Am. J. Physiol.* 254 F114–F120 3337241 10.1152/ajprenal.1988.254.1.F114

[B28] Chesler M. (2003). Regulation and modulation of pH in the brain. *Physiol. Rev.* 83 1183–1221 14506304 10.1152/physrev.00010.2003

[B29] Damkier H. H. Aalkjaer C. Praetorius J. (2010). Na^+^-dependent HCO_3_^-^ import by the slc4a10 gene product involves Cl^-^ export. *J. Biol. Chem.* 285 26998–27007 10.1074/jbc.M110.108712 20566632 PMC2930699

[B30] Dorwart M. R. Shcheynikov N. Yang D. Muallem S. (2008). The solute carrier 26 family of proteins in epithelial ion transport. *Physiology (Bethesda)* 23 104–114 10.1152/physiol.00037.2007 18400693

[B31] Ebihara S. Shirato K. Harata N. Akaike N. (1995). Gramicidin-perforated patch recording: GABA response in mammalian neurones with intact intracellular chloride. *J. Physiol.* 484 (Pt 1) 77–86 7541464 10.1113/jphysiol.1995.sp020649 PMC1157923

[B32] Fagiolini M. Hensch T. K. (2000). Inhibitory threshold for critical-period activation in primary visual cortex. *Nature* 404 183–186 10.1038/35004582 10724170

[B33] Farrant M. Kaila K. (2007). The cellular, molecular and ionic basis of GABAA receptor signalling. *Prog. Brain Res.* 160 59–87 10.1016/S0079-6123(06)60005-8 17499109

[B34] Fatima-Shad K. Barry P. H. (1993). Anion permeation in GABA- and glycine-gated channels of mammalian cultured hippocampal neurons. *Proc. Biol. Sci.* 253 69–75 10.1098/rspb.1993.0083 7690484

[B35] Fujiwara-Tsukamoto Y. Isomura Y. Imanishi M. Fukai T. Takada M. (2007). Distinct types of ionic modulation of GABA actions in pyramidal cells and interneurons during electrical induction of hippocampal seizure-like network activity. *Eur. J. Neurosci.* 25 2713–2725 10.1111/j.1460-9568.2007.05543.x 17459104

[B36] Galietta L. J. Haggie P. M. Verkman A. S. (2001). Green fluorescent protein-based halide indicators with improved chloride and iodide affinities. *FEBS Lett.* 499 220–224 10.1016/S0014-5793(01)02561-3 11423120

[B37] Garaschuk O. Linn J. Eilers J. Konnerth A. (2000). Large-scale oscillatory calcium waves in the immature cortex. *Nat. Neurosci.* 3 452–459 10.1038/74823 10769384

[B38] Giffard R. G. Lee Y. S. Ouyang Y. B. Murphy S. L. Monyer H. (2003). Two variants of the rat brain sodium-driven chloride bicarbonate exchanger (NCBE): developmental expression and addition of a PDZ motif. *Eur. J. Neurosci.* 18 2935–2945 10.1046/j.1460-9568.2003.03053.x 14656289

[B39] Gonzalez-Islas C. Chub N. Wenner P. (2009). NKCC1 and AE3 appear to accumulate chloride in embryonic motoneurons. *J. Neurophysiol.* 101 507–518 10.1152/jn.90986.2008 19036864 PMC2657071

[B40] Graber M. L. Dilillo D. C. Friedman B. L. Pastoriza-Munoz E. (1986). Characteristics of fluoroprobes for measuring intracellular pH. *Anal. Biochem.* 156 202–212 10.1016/0003-2697(86)90174-0 3740410

[B41] Gulacsi A. Lee C. R. Sik A. Viitanen T. Kaila K. Tepper J. M. (2003). Cell type-specific differences in chloride-regulatory mechanisms and GABAA receptor-mediated inhibition in rat substantia nigra. *J. Neurosci.* 23 8237–8246 12967985 10.1523/JNEUROSCI.23-23-08237.2003 PMC6740695

[B42] Hensch T. K. Fagiolini M. Mataga N. Stryker M. P. Baekkeskov S. Kash S. F. (1998). Local GABA circuit control of experience-dependent plasticity in developing visual cortex. *Science* 282 1504–1508 10.1126/science.282.5393.1504 9822384 PMC2851625

[B43] Hentschke M. Wiemann M. Hentschke S. Kurth I. Hermans-Borgmeyer I. Seidenbecher T. (2006). Mice with a targeted disruption of the Cl^-^/HCO_3_^-^ exchanger AE3 display a reduced seizure threshold. *Mol. Cell. Biol.* 26 182–191 10.1128/MCB.26.1.182-191.2006 16354689 PMC1317631

[B44] Hilgen G. Huebner A. K. Tanimoto N. Sothilingam V. Seide C. Garrido M. G. (2012). Lack of the sodium-driven chloride bicarbonate exchanger NCBE impairs visual function in the mouse retina. *PLoS ONE *7:e46155. 10.1371/journal.pone.0046155 PMC3467262 23056253

[B45] Horn R. Marty A. (1988). Muscarinic activation of ionic currents measured by a new whole-cell recording method. *J. Gen. Physiol.* 92 145–159 10.1085/jgp.92.2.145 2459299 PMC2228899

[B46] Hübner C. A. Hentschke M. Jacobs S. Hermans-Borgmeyer I. (2004). Expression of the sodium-driven chloride bicarbonate exchanger NCBE during prenatal mouse development. *Gene Expr. Patterns* 5 219–223 10.1016/j.modgep.2004.08.002 15567717

[B47] Hübner C. A. Stein V. Hermans-Borgmeyer I. Meyer T. Ballanyi K. Jentsch T. J. (2001). Disruption of KCC2 reveals an essential role of K-Cl cotransport already in early synaptic inhibition. *Neuron* 30 515–524 10.1016/S0896-6273(01)00297-5 11395011

[B48] Illsley N. P. Verkman A. S. (1987). Membrane chloride transport measured using a chloride-sensitive fluorescent probe. *Biochemistry* 26 1215–1219 10.1021/bi00379a002 3567167

[B49] Inglefield J. R. Schwartz-Bloom R. D. (1997). Confocal imaging of intracellular chloride in living brain slices: measurement of GABAA receptor activity. *J. Neurosci. Methods* 75 127–135 10.1016/S0165-0270(97)00054-X 9288644

[B50] Irie T. Hara M. Yasukura T. Minamino M. Omori K. Matsuda H. (1998). Chloride concentration in cultured hippocampal neurons increases during long-term exposure to ammonia through enhanced expression of an anion exchanger. *Brain Res.* 806 246–256 10.1016/S0006-8993(98)00700-8 9739146

[B51] Jacobs S. Ruusuvuori E. Sipila S. T. Haapanen A. Damkier H. H. Kurth I. (2008). Mice with targeted Slc4a10 gene disruption have small brain ventricles and show reduced neuronal excitability. *Proc. Natl. Acad. Sci. U.S.A.* 105 311–316 10.1073/pnas.0705487105 18165320 PMC2224208

[B52] Kaila K. Lamsa K. Smirnov S. Taira T. Voipio J. (1997). Long-lasting GABA-mediated depolarization evoked by high-frequency stimulation in pyramidal neurons of rat hippocampal slice is attributable to a network-driven, bicarbonate-dependent K^+^ transient. *J. Neurosci.* 17 7662–7672 9315888 10.1523/JNEUROSCI.17-20-07662.1997 PMC6793904

[B53] Kaila K. Voipio J. (1987). Postsynaptic fall in intracellular pH induced by GABA-activated bicarbonate conductance. *Nature* 330 163–165 10.1038/330163a0 3670401

[B54] Katz L. C. Shatz C. J. (1996). Synaptic activity and the construction of cortical circuits. *Science* 274 1133–1138 10.1126/science.274.5290.1133 8895456

[B55] Kim Y. Trussell L. O. (2009). Negative shift in the glycine reversal potential mediated by a Ca^2^^+^- and pH-dependent mechanism in interneurons. *J. Neurosci.* 29 11495–11510 10.1523/JNEUROSCI.1086-09.2009 19759298 PMC2894626

[B56] Kirmse K. Kirischuk S. (2006). Ambient GABA constrains the strength of GABAergic synapses at Cajal–Retzius cells in the developing visual cortex. *J. Neurosci.* 26 4216–4227 10.1523/JNEUROSCI.0589-06.2006 16624942 PMC6674013

[B57] Kirmse K. Witte O. W. Holthoff K. (2010). GABA depolarizes immature neocortical neurons in the presence of the ketone body ss-hydroxybutyrate. *J. Neurosci.* 30 16002–16007 10.1523/JNEUROSCI.2534-10.2010 21106838 PMC6633760

[B58] Kirmse K. Witte O. W. Holthoff K. (2011). GABAergic depolarization during early cortical development and implications for anticonvulsive therapy in neonates. *Epilepsia* 52 1532–1543 10.1111/j.1528-1167.2011.03128.x 21668443

[B59] Kobayashi S. Morgans C. W. Casey J. R. Kopito R. R. (1994). AE3 anion exchanger isoforms in the vertebrate retina: developmental regulation and differential expression in neurons and glia. *J. Neurosci.* 14 6266–6279 7931579 10.1523/JNEUROSCI.14-10-06266.1994 PMC6576973

[B60] Kopito R. R. Lee B. S. Simmons D. M. Lindsey A. E. Morgans C. W. Schneider K. (1989). Regulation of intracellular pH by a neuronal homolog of the erythrocyte anion exchanger. *Cell* 59 927–937 10.1016/0092-8674(89)90615-6 2686841

[B61] Kulik A. Nishimaru H. Ballanyi K. (2000). Role of bicarbonate and chloride in GABA- and glycine-induced depolarization and [Ca^2^^+^]_i_ rise in fetal rat motoneurons in situ. *J. Neurosci.* 20 7905–7913 11050110 10.1523/JNEUROSCI.20-21-07905.2000 PMC6772719

[B62] Kuner T. Augustine G. J. (2000). A genetically encoded ratiometric indicator for chloride: capturing chloride transients in cultured hippocampal neurons. *Neuron* 27 447–459 10.1016/S0896-6273(00)00056-8 11055428

[B63] Kyrozis A. Reichling D. B. (1995). Perforated-patch recording with gramicidin avoids artifactual changes in intracellular chloride concentration. *J. Neurosci. Methods* 57 27–35 10.1016/0165-0270(94)00116-X 7540702

[B64] Leinekugel X. Medina I. Khalilov I. Ben-Ari Y. Khazipov R. (1997). Ca^2^^+^ oscillations mediated by the synergistic excitatory actions of GABA(A) and NMDA receptors in the neonatal hippocampus. *Neuron* 18 243–255 10.1016/S0896-6273(00)80265-2 9052795

[B65] Lindau M. Fernandez J. M. (1986). IgE-mediated degranulation of mast cells does not require opening of ion channels. *Nature* 319 150–153 10.1038/319150a0 2417125

[B66] Luhmann H. J. Prince D. A. (1991). Postnatal maturation of the GABAergic system in rat neocortex. *J. Neurophysiol.* 65 247–263 1673153 10.1152/jn.1991.65.2.247

[B67] Majumdar D. Bevensee M. O. (2010). Na-coupled bicarbonate transporters of the solute carrier 4 family in the nervous system: function, localization, and relevance to neurologic function. *Neuroscience* 171 951–972 10.1016/j.neuroscience.2010.09.037 20884330 PMC2994196

[B68] Marandi N. Konnerth A. Garaschuk O. (2002). Two-photon chloride imaging in neurons of brain slices. *Pflugers Arch.* 445 357–365 10.1007/s00424-002-0933-7 12466938

[B69] Markova O. Mukhtarov M. Real E. Jacob Y. Bregestovski P. (2008). Genetically encoded chloride indicator with improved sensitivity. *J. Neurosci. Methods* 170 67–76 10.1016/j.jneumeth.2007.12.016 18279971

[B70] Mason M. J. Simpson A. K. Mahaut-Smith M. P. Robinson H. P. (2005). The interpretation of current-clamp recordings in the cell-attached patch-clamp configuration. *Biophys. J.* 88 739–750 10.1529/biophysj.104.049866 15516522 PMC1305049

[B71] McMilin K. D. Reiss J. A. Brown M. G. Black M. H. Buckmaster D. A. Durum C. T. (1998). Clinical outcomes of four patients with microdeletion in the long arm of chromosome 2. *Am. J. Med. Genet.* 78 36–43 10.1002/(SICI)1096-8628(19980616)78:1<36::AID-AJMG8>3.0.CO;2-J 9637421

[B72] McMurtrie H. L. Cleary H. J. Alvarez B. V. Loiselle F. B. Sterling D. Morgan P. E. (2004). The bicarbonate transport metabolon. *J. Enzyme Inhib. Med. Chem.* 19 231–236 10.1080/14756360410001704443 15499994

[B73] Minlebaev M. Ben-Ari Y. Khazipov R. (2007). Network mechanisms of spindle-burst oscillations in the neonatal rat barrel cortex in vivo. *J. Neurophysiol.* 97 692–700 10.1152/jn.00759.2006 17093125

[B74] Mohajerani M. H. Cherubini E. (2005). Spontaneous recurrent network activity in organotypic rat hippocampal slices. *Eur. J. Neurosci.* 22 107–118 10.1111/j.1460-9568.2005.04198.x 16029200

[B75] Morita K. Tsumoto K. Aihara K. (2005). Possible effects of depolarizing GABAA conductance on the neuronal input-output relationship: a modeling study. *J. Neurophysiol.* 93 3504–3523 10.1152/jn.00988.2004 15689391

[B76] Morita K. Tsumoto K. Aihara K. (2006). Bidirectional modulation of neuronal responses by depolarizing GABAergic inputs. *Biophys. J.* 90 1925–1938 10.1529/biophysj.105.063164 16387774 PMC1386773

[B77] Mueller A. L. Taube J. S. Schwartzkroin P. A. (1984). Development of hyperpolarizing inhibitory postsynaptic potentials and hyperpolarizing response to gamma-aminobutyric acid in rabbit hippocampus studied in vitro. *J. Neurosci.* 4 860–867 6707735 10.1523/JNEUROSCI.04-03-00860.1984 PMC6564832

[B78] Mukhtarov M. Liguori L. Waseem T. Rocca F. Buldakova S. Arosio D. (2013). Calibration and functional analysis of three genetically encoded Cl^-^/pH sensors. *Front. Mol. Neurosci. *6:9. 10.3389/fnmol.2013.00009 PMC3629305 23616745

[B79] Owens D. F. Boyce L. H. Davis M. B. Kriegstein A. R. (1996). Excitatory GABA responses in embryonic and neonatal cortical slices demonstrated by gramicidin perforated-patch recordings and calcium imaging. *J. Neurosci.* 16 6414–6423 8815920 10.1523/JNEUROSCI.16-20-06414.1996 PMC6578913

[B80] Park M. Li Q. Shcheynikov N. Zeng W. Muallem S. (2004). NaBC1 is a ubiquitous electrogenic Na^+^-coupled borate transporter essential for cellular boron homeostasis and cell growth and proliferation. *Mol. Cell.* 16 331–341 10.1016/j.molcel.2004.09.030 15525507

[B81] Parker M. D. Boron W. F. Tanner M. J. A. (2002). Characterization of human `AE4' as an electroneutral, sodium-dependent bicarbonate transporter. *FASEB J.* 16 A796

[B82] Parker M. D. Bouyer P. Daly C. M. Boron W. F. (2008a). Cloning and characterization of novel human SLC4A8 gene products encoding Na^+^-driven Cl^-^/HCO_3_^-^ exchanger variants NDCBE-A, -C, and -D. *Physiol. Genomics* 34 265–276 10.1152/physiolgenomics.90259.2008 18577713 PMC2519961

[B83] Parker M. D. Musa-Aziz R. Rojas J. D. Choi I. Daly C. M. Boron W. F. (2008b). Characterization of human SLC4A10 as an electroneutral Na/HCO_3_ cotransporter (NBCn2) with Cl^-^ self-exchange activity. *J. Biol. Chem.* 283 12777–12788 10.1074/jbc.M707829200 18319254 PMC2442331

[B84] Perkins K. L. Wong R. K. (1996). Ionic basis of the postsynaptic depolarizing GABA response in hippocampal pyramidal cells. *J. Neurophysiol.* 76 3886–3894 8985886 10.1152/jn.1996.76.6.3886

[B85] Pfeffer C. K. Stein V. Keating D. J. Maier H. Rinke I. Rudhard Y. (2009). NKCC1-dependent GABAergic excitation drives synaptic network maturation during early hippocampal development. *J. Neurosci.* 29 3419–3430 10.1523/JNEUROSCI.1377-08.2009 19295148 PMC6665272

[B86] Rae J. Cooper K. Gates P. Watsky M. (1991). Low access resistance perforated patch recordings using amphotericin B. *J. Neurosci. Methods* 37 15–26 10.1016/0165-0270(91)90017-T 2072734

[B87] Raley-Susman K. M. Sapolsky R. M. Kopito R. R. (1993). Cl^-^/HCO_3_^-^ exchange function differs in adult and fetal rat hippocampal neurons. *Brain Res.* 614 308–314 10.1016/0006-8993(93)91049-X 8348323

[B88] Rheims S. Minlebaev M. Ivanov A. Represa A. Khazipov R. Holmes G. L. (2008). Excitatory GABA in rodent developing neocortex in vitro. *J. Neurophysiol.* 100 609–619 10.1152/jn.90402.2008 18497364

[B89] Rink T. J. Tsien R. Y. Pozzan T. (1982). Cytoplasmic pH and free Mg^2^^+^ in lymphocytes. *J. Cell Biol.* 95 189–196 10.1083/jcb.95.1.189 6815204 PMC2112339

[B90] Rivera C. Voipio J. Payne J. A. Ruusuvuori E. Lahtinen H. Lamsa K. (1999). The K^+^/Cl^-^ co-transporter KCC2 renders GABA hyperpolarizing during neuronal maturation. *Nature* 397 251–255 10.1038/16697 9930699

[B91] Romero M. F. Chen A. P. Parker M. D. Boron W. F. (2013). The SLC4 family of bicarbonate (HCO_3_^-^) transporters. *Mol. Aspects Med.* 34 159–182 10.1016/j.mam.2012.10.008 23506864 PMC3605756

[B92] Romero M. F. Fulton C. M. Boron W. F. (2004). The SLC4 family of HCO_3_^-^ transporters. *Pflugers Arch.* 447 495–509 10.1007/s00424-003-1180-2 14722772

[B93] Romero M. F. Henry D. Nelson S. Harte P. J. Dillon A. K. Sciortino C. M. (2000). Cloning and characterization of a Na^+^-driven anion exchanger (NDAE1). A new bicarbonate transporter*. J. Biol. Chem.* 275 24552–24559 10.1074/jbc.M003476200 10827195

[B94] Roos A. Boron W. F. (1981). Intracellular pH. *Physiol. Rev.* 61 296–434 7012859 10.1152/physrev.1981.61.2.296

[B95] Rust M. B. Alper S. L. Rudhard Y. Shmukler B. E. Vicente R. Brugnara C. (2007). Disruption of erythroid K–Cl cotransporters alters erythrocyte volume and partially rescues erythrocyte dehydration in SAD mice. *J. Clin. Invest.* 117 1708–1717 10.1172/JCI30630 17510708 PMC1866252

[B96] Ruusuvuori E. Huebner A. K. Kirilkin I. Yukin A. Blaesse P. Helmy M. (2013). Neuronal carbonic anhydrase VII provides GABAergic excitatory drive to exacerbate febrile seizures. *EMBO J.* 32 2275–2286 10.1038/emboj.2013.160 23881097 PMC3746197

[B97] Ruusuvuori E. Li H. Huttu K. Palva J. M. Smirnov S. Rivera C. (2004). Carbonic anhydrase isoform VII acts as a molecular switch in the development of synchronous gamma-frequency firing of hippocampal CA1 pyramidal cells. *J. Neurosci.* 24 2699–2707 10.1523/JNEUROSCI.5176-03.2004 15028762 PMC6729533

[B98] Sander T. Toliat M. R. Heils A. Leschik G. Becker C. Ruschendorf F. (2002). Association of the 867Asp variant of the human anion exchanger 3 gene with common subtypes of idiopathic generalized epilepsy. *Epilepsy Res.* 51 249–255 10.1016/S0920-1211(02)00152-3 12399075

[B99] Schwiening C. J. Boron W. F. (1994). Regulation of intracellular pH in pyramidal neurones from the rat hippocampus by Na^+^-dependent Cl–HCO_3_^-^ exchange. *J. Physiol.* 475 59–67 8189393 10.1113/jphysiol.1994.sp020049 PMC1160355

[B100] Seja P. Schonewille M. Spitzmaul G. Badura A. Klein I. Rudhard Y. (2012). Raising cytosolic Cl^-^ in cerebellar granule cells affects their excitability and vestibulo-ocular learning. *EMBO J.* 31 1217–1230 10.1038/emboj.2011.488 22252133 PMC3297995

[B101] Sinning A. Liebmann L. Kougioumtzes A. Westermann M. Bruehl C Hübner C. A. (2011). Synaptic glutamate release is modulated by the Na^+^-driven Cl^-^/HCO_3_^-^ exchanger Slc4a8. *J. Neurosci.* 31 7300–7311 10.1523/JNEUROSCI.0269-11.2011 21593314 PMC6622604

[B102] Sipila S. T. Schuchmann S. Voipio J. Yamada J. Kaila K. (2006). The cation-chloride cotransporter NKCC1 promotes sharp waves in the neonatal rat hippocampus. *J. Physiol.* 573 765–773 10.1113/jphysiol.2006.107086 16644806 PMC1779742

[B103] Staley K. J. Soldo B. L. Proctor W. R. (1995). Ionic mechanisms of neuronal excitation by inhibitory GABAA receptors. *Science* 269 977–981 10.1126/science.7638623 7638623

[B104] Stein V. Hermans-Borgmeyer I. Jentsch T. J Hübner C. A. (2004). Expression of the KCl cotransporter KCC2 parallels neuronal maturation and the emergence of low intracellular chloride. *J. Comp. Neurol.* 468 57–64 10.1002/cne.10983 14648690

[B105] Svichar N. Waheed A. Sly W. S. Hennings J. C. Hübner C. A. Chesler M. (2009). Carbonic anhydrases CA4 and CA14 both enhance AE3-mediated Cl^-^/HCO_3_^-^ exchange in hippocampal neurons. *J. Neurosci.* 29 3252–3258 10.1523/JNEUROSCI.0036-09.2009 19279262 PMC2757777

[B106] Thomas R. C. (1974). Intracellular pH of snail neurones measured with a new pH-sensitive glass mirco-electrode. *J. Physiol.* 238 159–180 4838803 10.1113/jphysiol.1974.sp010516 PMC1330868

[B107] Thomas R. C. (1977). The role of bicarbonate, chloride and sodium ions in the regulation of intracellular pH in snail neurones. *J. Physiol.* 273 317–338 23429 10.1113/jphysiol.1977.sp012096 PMC1353741

[B108] Tyzio R. Cossart R. Khalilov I. Minlebaev M. Hübner C. A. Represa A. (2006). Maternal oxytocin triggers a transient inhibitory switch in GABA signaling in the fetal brain during delivery. *Science* 314 1788–1792 10.1126/science.1133212 17170309

[B109] Tyzio R. Ivanov A. Bernard C. Holmes G. L. Ben-Ari Y. Khazipov R. (2003). Membrane potential of CA3 hippocampal pyramidal cells during postnatal development. *J. Neurophysiol.* 90 2964–2972 10.1152/jn.00172.2003 12867526

[B110] Tyzio R. Holmes G. L. Ben-Ari Y. Khazipov R. (2007). Timing of the developmental switch in GABA(A) mediated signaling from excitation to inhibition in CA3 rat hippocampus using gramicidin perforated patch and extracellular recordings. *Epilepsia* 48(Suppl.5) 96–105 10.1111/j.1528-1167.2007.01295.x 17910587

[B111] Vaughan-Jones R. D. (1979). Non-passive chloride distribution in mammalian heart muscle: micro-electrode measurement of the intracellular chloride activity. *J. Physiol.* 295 83–109 521996 10.1113/jphysiol.1979.sp012956 PMC1278788

[B112] Verheugen J. A. Fricker D. Miles R. (1999). Noninvasive measurements of the membrane potential and GABAergic action in hippocampal interneurons. *J. Neurosci.* 19 2546–2555 10087068 10.1523/JNEUROSCI.19-07-02546.1999 PMC6786065

[B113] Vilas G. L. Johnson D. E. Freund P. Casey J. R. (2009). Characterization of an epilepsy-associated variant of the human Cl^-^/HCO_3_^-^ exchanger AE3. *Am. J. Physiol. Cell Physiol.* 297 C526–C536 10.1152/ajpcell.00572.2008 19605733

[B114] Walker J. L. (1971). Ion specific liquid ion exchanger microelectrodes. *Anal. Chem.* 43 89A–93A 10.1021/ac60298a039

[B115] Wang C. Z. Yano H. Nagashima K. Seino S. (2000). The Na^+^-driven Cl^-^/HCO_3_^-^ exchanger. Cloning, tissue distribution, and functional characterization. *J. Biol. Chem.* 275 35486–35490 10.1074/jbc.C000456200 10993873

[B116] Wang D. D. Krueger D. D. Bordey A. (2003). GABA depolarizes neuronal progenitors of the postnatal subventricular zone via GABAA receptor activation. *J. Physiol.* 550 785–800 10.1113/jphysiol.2003.042572 12807990 PMC2343064

[B117] Yamada J. Okabe A. Toyoda H. Kilb W. Luhmann H. J. Fukuda A. (2004). Cl^-^ uptake promoting depolarizing GABA actions in immature rat neocortical neurones is mediated by NKCC1. *J. Physiol.* 557 829–841 10.1113/jphysiol.2004.062471 15090604 PMC1665166

[B118] Yuste R. Katz L. C. (1991). Control of postsynaptic Ca^2^^+^ influx in developing neocortex by excitatory and inhibitory neurotransmitters. *Neuron* 6 333–344 10.1016/0896-6273(91)90243-S 1672071

